# Region-Based Collision Avoidance Beaconless Geographic Routing Protocol in Wireless Sensor Networks

**DOI:** 10.3390/s150613222

**Published:** 2015-06-05

**Authors:** JeongCheol Lee, HoSung Park, SeokYoon Kang, Ki-Il Kim

**Affiliations:** 1 Department of Computer Science, University of California, Los Angeles, CA 90095, USA; E-Mail: jclee0333@gmail.com; 2 Department of Informatics, Research Center for Aerospace Parts Technology, Gyeongsang National University, Jinju 660-701, Korea; E-Mails: hspark0865@gmail.com (H.P.); syk@gnu.ac.kr (S.K.)

**Keywords:** region-based collision avoidance, beaconless geographic routing protocol, wireless sensor networks

## Abstract

Due to the lack of dependency on beacon messages for location exchange, the beaconless geographic routing protocol has attracted considerable attention from the research community. However, existing beaconless geographic routing protocols are likely to generate duplicated data packets when multiple winners in the greedy area are selected. Furthermore, these protocols are designed for a uniform sensor field, so they cannot be directly applied to practical irregular sensor fields with partial voids. To prevent the failure of finding a forwarding node and to remove unnecessary duplication, in this paper, we propose a region-based collision avoidance beaconless geographic routing protocol to increase forwarding opportunities for randomly-deployed sensor networks. By employing different contention priorities into the mutually-communicable nodes and the rest of the nodes in the greedy area, every neighbor node in the greedy area can be used for data forwarding without any packet duplication. Moreover, simulation results are given to demonstrate the increased packet delivery ratio and shorten end-to-end delay, rather than well-referred comparative protocols.

## 1. Introduction

The geographic routing protocol requires nodes to know the position of their neighbors for forwarding data, so each node periodically exchanges HELLO messages, including its position with its neighbors. However, periodical message exchange can be another overhead when considering the node's constraints in wireless sensor networks. Thus, in order to reduce such control overhead caused by these messages, called beacons, the beaconless geographic routing strategy has been recently studied in the literature.

Different from beacon-based geographic routing protocols, typical beaconless routing [1,2] has the following process: a sender broadcasts data to its neighbors, and only neighbors in the greedy forwarding area, closer neighbors to a destination, are eligible to become next-hop forwarding candidates. Then, only one neighbor is selected as a next-hop forwarder by a completely reactive method. To avoid collision among these neighbors, the sender includes a waiting function in the data packet, which is related to the distance between each receiving neighbor and the destination. Therefore, a node having the fastest expired timer, that is the closest node from the destination among the neighbors, will become a next-hop forwarder by itself and begin to send the received data by broadcasting, as the previous sender did. The rest of the neighbors overhear this message, so they could cancel their own timer and release the received data.

Besides the mentioned basic operation, we should consider abnormal situations. For example, if some of the neighbors in the greedy area cannot hear the message of other neighbors, because any two nodes may be possibly out of the radio range, it is hard to guarantee that every forwarding candidate overhears each other. This circumstance is very similar to the hidden terminal problem in the 802.11 wireless networks, and it might lead to a large number of packet duplications in such overhearing-based beaconless routing protocols, due to multiple winners among these neighbors. Therefore, in order to solve the packet duplication problem, previous studies [1,2] have tried to adopt a completely conservative approach, which reduces a forwarding candidate area to a restricted region. This approach limits the greedy forwarding area to only mutually-communicable nodes. It allows that only the nodes in the restricted forwarding area, which is nested in the greedy area, can participate in the timer-based forwarding contention.

This strategy is very simple and may be effective in uniformly- and densely-deployed WSNs, but forwarding opportunities can be lost in practical WSNs. In most of the applications of WSNs, very small and inexpensive sensor nodes are deployed to an field of interest by a plane in the air. Therefore, the network commonly becomes an irregular shape that has many partial network holes due to obstacles, such as buildings, lakes, *etc*. If such holes are in the restricted small forwarding area, finding a forwarding node would fail, even if there are appropriate candidates in the rest of the area.

**Figure 1:**
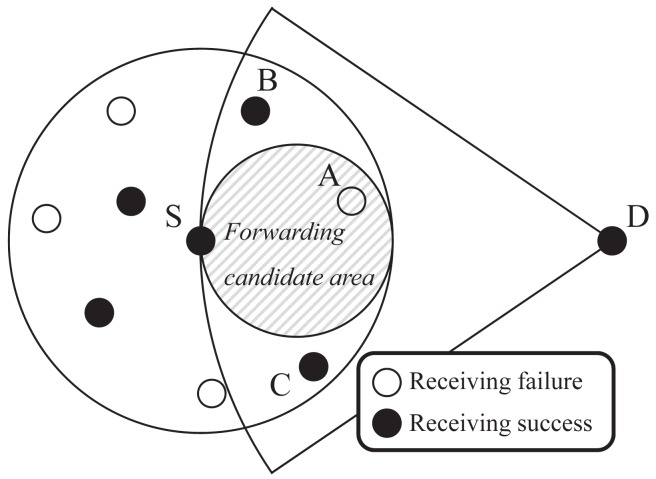
An example of next-hop forwarder selection failure in the restricted forwarding area-based beaconless routing protocol.

For example, as shown in Figure 1, the sender *S* broadcasts its data packet to its neighbors, including both the location information of the forwarding candidate area (dashed small circle area) from the sender and position of the destination *D*. Note that node in the forwarding candidate area tries to wait differently according to its own timer, related to the distance between the destination and itself, while nodes in the rest of the area drop the received packet from *S*. However, in this case, there are no nodes that successfully receive the sender's broadcast data in the restricted forwarding candidate area due to both the network holes and transmission failure. Although there exist appropriate candidates in the rest of the area (Nodes *B* and *C*), the sender *S* has to send the data packet again or change its routing mode from the greedy mode to the recovery mode. Unfortunately, the neighbors cannot be guaranteed to successfully receive the data packet again at the next time due to the error-prone nature of wireless links. Furthermore, if the routing mode is changed, the protocol requires a number of control messages and wastes much node energy, because it has to get the positions of all neighbors to detour the holes. These unnecessary and redundant control messages may reduce the entire network performance.

Therefore, in order to both increase forwarding opportunities and also prevent packet duplication due to the hidden problem, we propose a semi-distributed beaconless geographic routing protocol, which gives a priority-based delay function for the forwarding node selection into each region in the sub-areas of the greedy forwarding area. In the proposed protocol, nodes in the high prioritized region that are mutually-communicable with each other find a next-hop forwarder immediately in a fully-distributed manner after receiving a sender's broadcast data. On the other hand, nodes in the low prioritized region have to wait until the contention of the high prioritized region is done. Namely, our protocol has two phases for the contention process. After the primary contention process, the low prioritized region begins the secondary contention process for finding an appropriate next-hop forwarder. Such a temporal difference easily allows a sender to know whether there is a partial hole in the high prioritized region without any control messages. Consequently, this information could give the sender an opportunity to select an appropriate next-hop forwarder in the rest of the area without the packet duplication problem. In other words, instead of the primary contention, only the secondary contention process works with an acknowledgement-based forwarding rule in order to prevent packet duplication due to the hidden problem. Experimental results show that the proposed protocol has better performance than previous restricted region-based fully-distributed beaconless routing protocols in terms of the data throughput, the total number of packets, the packet delivery ratio and the end-to-end delay.

The rest of this paper is organized as follows. Section 2 shows related work for existing beaconless geographic routing protocols. We describe the proposed protocol in detail in Section 3. Experimental results are given for evaluating the performance of the proposed protocol in the Section 4. Section 5 concludes this paper.

## 2. Related Work

Geographic routing has been considered as an efficient and scalable routing protocol, since it exploits pure location information to route data packets to a destination instead of global network topology information in error-prone wireless sensor networks. This scheme requires three kinds of location information: the position of the current node holding the data packet, the position of its neighbor nodes and the position of the destination.

The basic idea of geographic routing is that the current node compares the Euclidean distance from each neighbor to the destination and selects the closest neighbor to the destination as a next-hop forwarding node. Finally, the current node sends the data packet to the closest neighbor, and this process is repeated until the packet arrives at the destination. This is called greedy routing. However, if there is no neighbor closer to the destination than the current node, it changes its routing mode from the greedy routing mode into the perimeter routing mode, which is based on the application of the right-hand rule over the planarized vision of the underlying graph. In order to realize the location information of neighbor nodes before routing, every sensor node in the network periodically should exchange the control message, including its identifier and its location information, called the beacon. Beacons are not flooded across the whole network, but the overhead can still be excessive and nonproductive [3]. Namely, regardless of the data routing, it leads to unnecessary energy consumption, interference, collisions and/or congestion problems.

To avoid this issue generated by the beacons, many beaconless geographic routing protocols [1–19] have been proposed in the literature. The basic idea of beaconless routing is to reactively discover the location information of neighbor nodes to select (or to be selected) the next-hop forwarding node when routing data packets. It can be divided into two different schemes according to the forwarding node selection method: the fully-distributed method and the handshake-based method.

The first one is a fully-distributed beaconless geographic routing scheme [1,2,4,5]. In this scheme, a forwarding node is automatically selected among neighbors that successfully receive a sender's data by combining the distance-based timer and overhearing techniques. Namely, neighbors select a forwarding node themselves, instead of a node currently holding data.

The beaconless routing algorithm [1] (BLR) selects a forwarding node among neighbors having information neither about their position nor even about their existence. A node currently holding a data packet just broadcasts the data packet, and the closest neighbor to the destination will resend the packet, because its timer will expire first. The rest of the neighbors that successfully overhear the broadcast message cancel their timers. This process is repeated until the data packet reaches the destination. For a recovery strategy, in case there is no node in the forwarding candidate area, the current node broadcasts a short request message, and all neighbor nodes reply with a packet indicating their location information. Therefore, the current node could build the planar graph and exploit the recovery scheme of Greedy-Face-Greedy(GFG) [20] to determine an appropriate forwarding node in the perimeter mode.

The blind geographic routing [2] (BGR) protocol is an improved version of the previous one, BLR. The forwarding process is very similar to BGR, but the recovery process is quite different. For a recovery strategy, BGR turns the forwarding area by 60 degrees to the left or to the right by broadcasting an area change message. However, it might cause packet failures when nodes try to forward almost simultaneously. Therefore, BGR introduces the Avoidance of Simultaneous Forwarding (ASF) technique in which the number of hops is stored in the packet header. BLR and BGR have to avoid duplications due to neighbors that cannot overhear broadcast message, so they exploit a restricted forwarding area for which only the mutually-communicable nodes can be the forwarding candidate nodes.

However, this strategy is unnecessarily conservative; therefore, it might fail at finding a forwarding node and change the routing mode into the perimeter mode too fast, even if there exist appropriate forwarding candidates in the rest of the greedy forwarding area.

Recently, D. Rosario, *et al.* have introduced Link quality and Geographical-aware OR protocol (LINGO) [4] for video transmission, which exploits a cross-layer link quality and geographical-aware beaconless opportunistic routing protocol. LINGO combines link quality, geographic information and energy for routing decisions under dynamic topology changes. The extension version of LINGO, XLINGO [5], has been proposed by the same authors, in order to deal with node and link failures, as well as route failures by combining a set of cross-layer and human-related parameters for routing decisions, such as packet delivery ratio, queue length, link quality, geographic locations and residual energy.

Both LINGO and XLINGO can provide realistic and practical dynamic forwarding delay (DFD) for beaconless routing, but they cannot be directly adopted to wireless sensor networks due to their strict route creation for the hidden terminal problem.

The second one is a handshake-based beaconless geographic routing scheme [3,6–15]. In this scheme, a sender currently holding data sends an Ready-to-Send(RTS) message (request) to get the location information of the neighbors. Neighbors that successfully receive the request packet send a Clear-to-Send (CTS) message (response) to the sender according to the distance-based timer. Finally, the sender selects an appropriate forwarding node and sends the data packet to the node.

The contention-based forwarding [6] (CBF) protocol proposes three different suppression strategies, which vary with respect to forwarding efficiency and suppression characteristics. These strategies are able to make a sender select a next hop forwarding node without beacons by using the RTS/CTS/DATA approach. The Beacon-less On Demand Strategy (BOSS) [3] protocol includes a data packet in the RTS packet in order to improve the probability of data forwarding. Since a data packet has commonly a bigger packet size than other control packets, such as a traditional RTS/CTS packet, a sender firstly broadcasts an RTS packet, including the data packet, to all of its neighbors, and they respond with a CTS packet to the sender by the distance-based timer. After that, the sender sends only a small-sized ACK message to an appropriate forwarding node.

This BOSS protocol was well analyzed and evaluated by carrying out an empirical evaluation in a real testbed in order to show the correctness of our simulation results in [7]. Based on these research results, the authors reviewed some of the latest proposals in the field of beaconless geographic routing and introduced the main design challenges and alternatives in [8]. These research efforts led the development of the self-protected beaconless geographic routing (SBGR) protocol [9]. The main objective of SBGR is to take malicious nodes into account and to analyze the effects of insider attacks in beaconless geographic routing protocols.

Another research work for the handshake-based beaconless geographic routing protocol was conducted to meet special requirements. Ruhrup [10] has shown that delivery is guaranteed by beaconless forwarder planarization (BFP) and the new circlunar neighborhood graph (CNG). Furthermore, simulation results were presented to show similar message complexities in the average case. This research is extended to include the recovery scheme using only three messages (RTS, CTS and DATA) per forwarding step in [11].Next, the cross-layer approach was taken for beaconless geographical routing. Based on location knowledge and contention processes, the proposed cross-layer protocol, CoopGeo [12], aims at providing an efficient, distributed approach to select next hops and optimal relays. Furthermore, it introduces asymmetric encrypting of data packets to protect from malicious node attacks [13]. Energy-efficient beaconless geographic routing (EBGR) [14] was proposed to provide loop-free, fully-stateless, energy-efficient sensor-to-sink routing at a low communication overhead without the help of prior neighborhood knowledge by prohibiting the unsuitable neighbors from participating in the relay contention procedure. Context-aware Adaptive Opportunistic Routing (CAOR) [15] exploits multiple cross-layer context information, such as link quality, geographic progress, energy and mobility for mobile *ad hoc* network routing.

However, these handshake-based routing protocols cannot be directly applied to the error-prone wireless sensor networks because of their strict limitations. They always require three types of successful message exchanges between a sender and neighbors for each data forwarding in a hop: data packet, response packet and acknowledgment packet. Furthermore, they have a significant delay problem in reactively discovering the location information of neighbors. These limitations causes them to not be able to be used in most real-time sensor applications, such as intruder detection and fire alert systems.

## 3. Region-Based Collision Avoidance Beaconless Geographic Routing

### 3.1. System and Network Model

In the proposed protocol, the greedy forwarding area is divided into two sub-areas: hidden-less area and the hidden area. The hidden-less area is similar to the restricted forwarding area of the previous protocols by including only mutually-communicable nodes. However, unlike the previous protocols, the position of the hidden-less area can be moved within the greedy forwarding area by a learning mechanism. In order to switch the hidden-less area with simple calculation, we choose a 60-radian degree area for the hidden-less area, which is a radial region that includes a 30-radian degree area around the line connecting the sender and the destination on both sides. Except for the hidden-less area, we call the rest of the area the hidden area. Note that all nodes in the greedy forwarding area have been given data packets from the previous sender at the same time, but their relaying or answering times have to be different from each other in order to prevent collisions. Therefore, we exploit the modified waiting function, which is based on both the distance from the destination (closer node from the destination having a shorter timer) and the priority value related to its geographic position in the greedy forwarding area (higher prioritized node having a shorter timer).

Since beaconless geographic routing protocols mostly rely on the geographic greedy forwarding strategy, we assume that every sensor node in the network could know its own geographic information by GPS devices or using other localization algorithms [21], like the other beaconless geographic routing protocols. We also assume that a data source node could get the location of the destination by using the sink location service protocol [22,23] or its global flooding.

### 3.2. Overview of the Proposed Protocol

This section shows an overview of the proposed protocol in order to support understanding. We provide the policy of the protocol, flowcharts of both the sender and receiver side and figures for each routing mode: greedy routing mode and perimeter routing mode.

**Figure 2:**
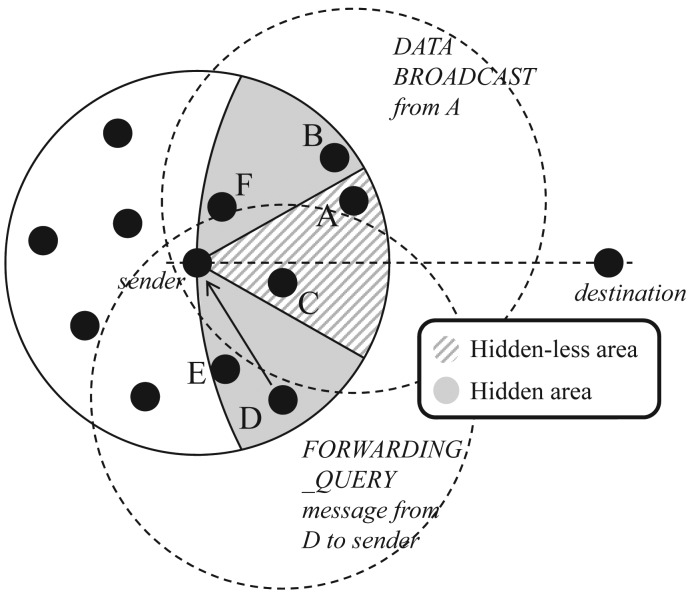
Data forwarding in the proposed protocol.

As shown in Figure 2, a sender broadcasts its data to its neighbors, so all nodes in the greedy forwarding area can receive the sender's data. The data packet contains the original message, the position of the sender and the destination, the maximum waiting time, the region information for the hidden-less area and routing mode information. If the routing mode of a packet is the perimeter mode, the position of a “stuck node” is additionally included in the packet header in order to prevent routing loop problems. This is described in detail in the recovery scheme Section. Each neighbor in the greedy forwarding area could realize whether it is in the hidden-less area or not. In this case, only the Nodes *A, B, C, D, E* and *F* have been receiving the data successfully among neighbors in the greedy forwarding area. These nodes start to keep the received data in their memories. Among these nodes, only *A* and *C* are in a high prioritized hidden-less area (fan-shaped dashed region). After receiving the data, nodes in the hidden-less area get higher priority than the hidden area, and they immediately begin to find a next-hop forwarder. These nodes have their own timer related only to the distance from the destination by using a predefined maximum time of *T_max_* seconds. Therefore, in the hidden-less area, the closest Node *A* from the destination wakes up first, becomes a next-hop forwarder by itself and broadcasts the received data to its neighbors. Among all neighbors in the greedy forwarding area, nodes that overhear this broadcast data release their timer and received data. However, if the node density is extremely high, this forwarding message might be generated simultaneously among neighbors, because timers expire almost concurrently. This might lead to many collisions, so the proposed protocol exploits the distance- and angle-based collision avoiding scheme (DACAS). This scheme is described in detail in the next section.

**Figure 3:**
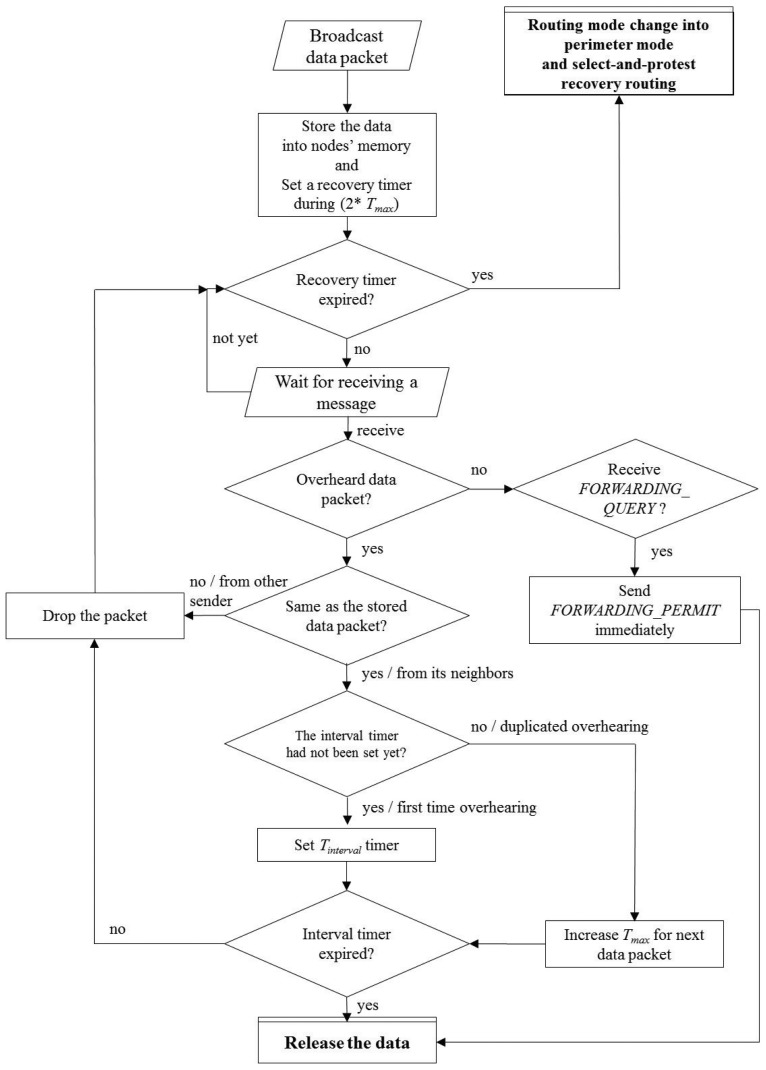
The flowchart of the sender side.

**Figure 4:**
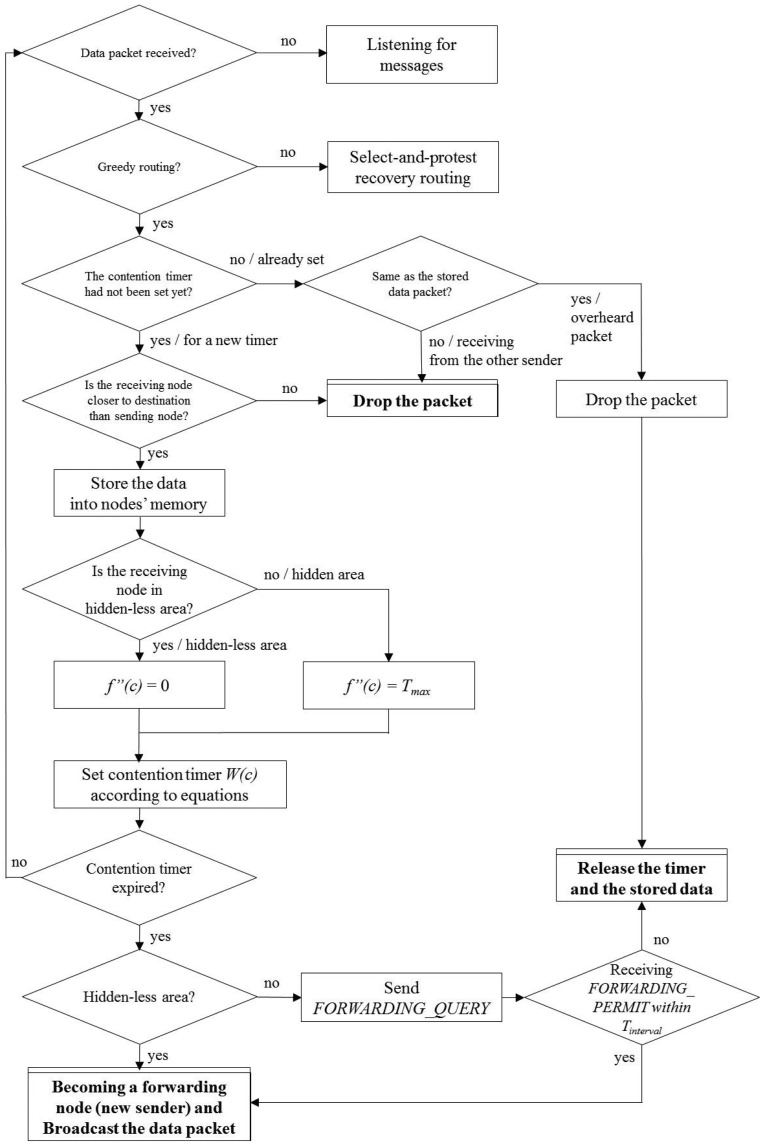
The flowchart of the receiver side.

On the other hand, nodes in the hidden area set their own timer as a sum of the distance-based value and the *T_max_*. Namely, every node in the hidden area has to wait during *T_max_* seconds first, then begins its distance-based timer. If the timer is expired, the node in the hidden area sends a *FORWARDING_QUERY* message to the sender. If there are no nodes that successfully rebroadcast the data in the hidden-less area, the sender replies the *FORWARDING_PERMIT* message to the node immediately. After that, the node becomes a next-hop forwarding node and broadcasts the received data to its neighbors. Except this node, other neighbors release their timers and receive data when they overhear the *FORWARDING_PERMIT* message. In this figure, Node *A* in the hidden-less area becomes a next-hop forwarding node by itself. The other nodes, *B, C* and *F*, that can overhear a broadcast data from *A* give up their contention process for becoming the next-hop forwarder. Since Nodes *D* and *E* are out of the transmission range from *A*, they cannot overhear the broadcast data message. When the timer of Node *D* is expired, it sends *FORWARDING_QUERY* messages to the sender *S*. However, the sender *S* already overheard the broadcast data from *A*, so *S* ignores the message from Node *D*. Furthermore, Node *E* can release its timer when it overhears a *FORWARDING_QUERY* message from Node *D*. Both Figures 3 and 4 show the flow of the routing decision of both the sender side and the receiver side.

### 3.3. Distance- and Angle-Based Collision Avoiding Scheme

The proposed protocol uses two different types of timers in order to avoid collisions due to simultaneous broadcasting among neighbors: *T_max_* and *T_interval_*. The values of these timers are given by the application during the network initializing stage.

When a sender broadcasts its data to its neighbors, neighbors which are closer to the destination than the sender (in the greedy forwarding area) set their own timer as the following equation:

(1) $$W\left(c\right)=f\left(c\right)+f^{\prime }\left(c\right)+f^{\prime \prime }\left(c\right)$$

where *W*(*c*) represents the total waiting time (ms) for the current node between zero and 2*T_max_. Once a node sets its timer by a packet broadcasting, the timer cannot be reset or added by other broadcasting packets. During the timer process, packets would be added into the queue stack of the node in order to avoid collisions from multiple sources and destinations.

**Figure 5:**
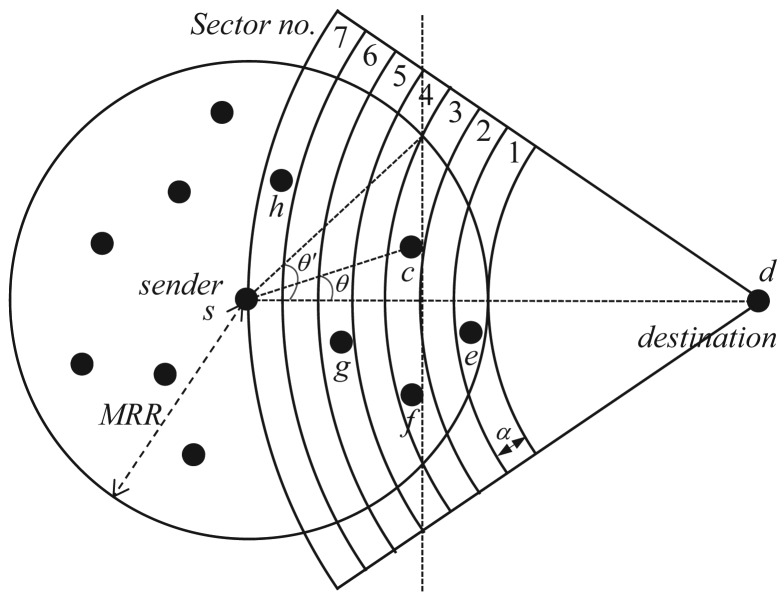
Sector area division for data forwarding. A small number of sectors means a short timer. *e, g* and *h* are Sectors 1, 5 and 7, respectively, whereas *c* and *f* are Sector 3.

As shown in Figure 5, the greedy area of a sender, which is closer to the destination than the sender, can be divided into multiple sector areas. Each sector is made by using the *Maximum_Radio_Range (MRR)* and the sector size *α* given by the application. The function *f* represent the waiting time for each sector, and the *f′* represents the local waiting time for each node in a sector according to its position in the sector. The *f″* represents the priority time delay. If Node *C* is in the hidden-less area, then the function *f″* returns zero. Otherwise, it returns *T_max_*. For the function *f*, it uses the following equation:

(2) $$f\left(c\right)=T_{\text{max}}\cdot \frac{\beta +\text{MRR}}{\text{MRR}}$$

where *dist*(*a, b*) represents the Euclidean distance between the position of Nodes *a* and *b. β* can be presented as the following equation:

(3) $$\beta =\alpha \cdot \left\lfloor \frac{\text{dist}\left(c,d\right)-\text{dist}\left(s,d\right)}{\alpha }\right\rfloor$$

where the values *s, c* and *d* are the geographic location of the sending node that broadcasts the data packet, the current node that successfully receives the data packet and the destination node, respectively. According to the sector size *α*, similarly-located sensor nodes have the same waiting time for the sector. The function *f′* can be presented as the following equation:

(4) $$f^{\prime }\left(c\right)=T_{\text{max}}\cdot \frac{\gamma \cdot \alpha }{\text{MRR}}$$

where the value γ falls between zero and one, according to the angle ratio of a sensor node in a sector area. A lower γ means that the node is closer to the line that is connecting the sender and the destination. Namely, the closest node from the source-destination line has the shortest timer in each sector area. The value γ for each node can be calculated as the following expression:

(5) $$\gamma =\left|\frac{\Theta }{\Theta^{\prime }}\right|$$

where Θ is the angle from the line connecting the sender and the destination to the current node *c*, and Θ′ is the angle from the line connecting the sender and the destination to the maximum sector angle (MSA). In the greedy routing mode, Θ and Θ′ vary from −90 to 90. For example, as shown in Figure 5, Node *c* is in sector No. 3. The MSA value of the third sector is 43 degrees, and the angle of Node *c* is 17 degrees. In this case, this is about 0.4. Each MSA can be calculated with the sector size and two intersection points between the sender circle and the destination circle.

(6) $$\text{MSA}\left(\theta \right)=\delta +\cos^{-1}\left(\frac{r_{1}^{2}-r_{2}^{2}+D^{2}}{2r_{1}D}\right)$$

where *r*_1_ could be represented as MRR and *r*_2_ could be the radius from the destination to each sector area. It could be as:

(7) $$r_{2}=\text{dist}\left(s,d\right)-\text{MRR}+n\cdot \alpha^{2}$$

where *n* is the number of each sector area. *δ* and *D* are represented as follows:

(8) $$\begin{array}{cc} \delta =\tan^{-1}\frac{y_{d}-y_{s}}{x_{d}-x_{s}}, & D=\sqrt{{\left(x_{d}-x_{s}\right)}^{2}+{\left(y_{d}-y_{s}\right)}^{2}} \end{array}$$

Furthermore, every sensor node has another timer called *T_interval_* for the adoptive collision avoidance. After a sender node broadcasts the data packet, if the node realizes a collision among its neighbors by the MAC device or receives two or more packets from its neighbors within *T_interval_*, it determines that there might be a collision among neighbors and rebroadcasts data with increased *T_max_*.

### 3.4. Recovery Scheme

If greedy routing fails due to a local minimum, a data packet may be stuck at a node that has no neighbor nodes closer to the destination than the node. For the case of routing failures, a recovery scheme is required in order to guarantee data delivery. In the proposed protocol, a sender holding a data packet could be aware of the case of the local minimum when the sender node cannot receive either a *FORWARDING_QUERY* or an overhearddata packet, the same as the helddata packet within 2 × *T_max_*.

In this case, the sender changes its routing mode into perimeter mode from greedy mode and rebroadcasts the data packet, including the perimeter routing mode and the position of the sender as a stuck node in the header of the packet. Once the packet meets an intermediate node that is closer to the destination than the stuck node, it would change the routing mode into greedy routing again.

In the perimeter mode, *i.e.*, beaconless recovery mode, the proposed protocol exploits the select-and-protest principle [24] to avoid crossing edges, which might lead to routing loops [25]. It has been proven in [10] that the routing loop problem could be solved as long as the network is connected, since the angular relaying algorithm can always select the first edge of the Gabriel graph (GG) in the order of (counter-)clockwise.

For a detailed description, in the selection phase, neighbor nodes that are further from a destination than a stuck node and successfully receive the data packet could be selected as candidates to form a local planar subgraph in each hop. After that, in the protest phase, violating edges could be removed from the subgraph by itself, as well as *PROTEST_QUERY* from other nodes. Consequently, the face routing algorithm can be applied to select the forwarder.

**Figure 6:**
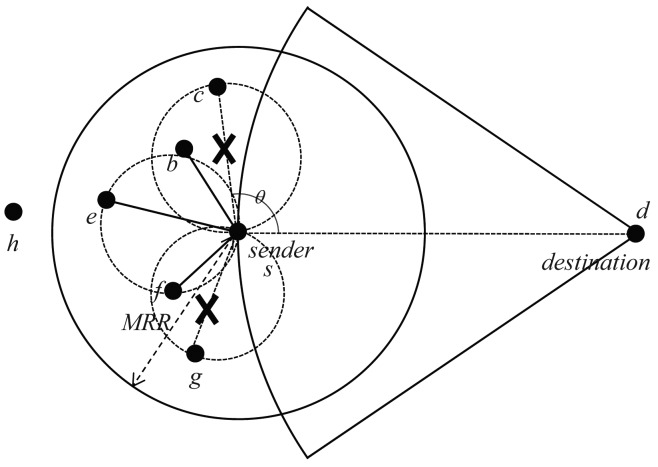
An example of the beaconless recovery forwarding scheme.

For example, as shown in Figure 6, *b, f* and *h* receive the *FORWARDING_QUERY* message from *c, g* and *e*, respectively. The *b* and *f* are in the proximity region of *c* and *g*, and *b* protest against *c*, as well as *f* protests against *g*. Therefore, the sender *S* could get a planar subgraph for face routing by eliminating violated edges. The selected next-hop forwarder would receive *FORWARDING_PERMIT* by the *s*.

## 4. Simulation Evaluation

We evaluate the performance of the proposed protocol in terms of the data throughput, the total number of packets, the packet delivery ratio and the end-to-end delay with well-known beaconless routing protocols in wireless sensor networks: BLR [1], BGR [2] and BOSS [3]. These protocols are selected according to the similarity with the proposed scheme.

### 4.1. Methodology and Metrics

We implement the four schemes by Qualnet Network Simulator 4.0 [26] and utilize IEEE 802.15.4 as the MAC protocol. The model of the sensor nodes is followed by the specification of MICA2 [27]. The transmission range of sensor nodes is set to 50 m. The simulation scenario is a 500 m × 500 m area in which a varying number of nodes (from 200 to 900 nodes) are deployed. To conduct a fair comparison, each protocol sets *T_max_* as 300 ms. For the proposed protocol, *T_interval_* is set to 10 ms. For each scenario, the results presented here are the average of 10 separate simulation runs.

In order to evaluate the performance of the proposed protocol, we consider the following metrics. Furthermore, the 95% confidence intervals on the mean are computed.

- Data throughput: we evaluate the data throughput of the route from a source to the destination of each protocol by using the number of packets per second delivered.
- Hop counts: the number of hops of the path from the source to the destination.
- Total number of packets: this metric presents the total number of transmissions, including both data packets and control packets by sensor nodes.
- Total number of perimeter packets: this metric presents only the number of transmissions made during perimeter routing.
- Packet delivery ratio: this metric represents the ratio of the number of successfully reached data packets at the destination among generated data packets by the source.
- End-to-end delay: the average time for sending data packets from the source to the destination, including all kinds of delay.

### 4.2. Data Throughputs

**Figure 7:**
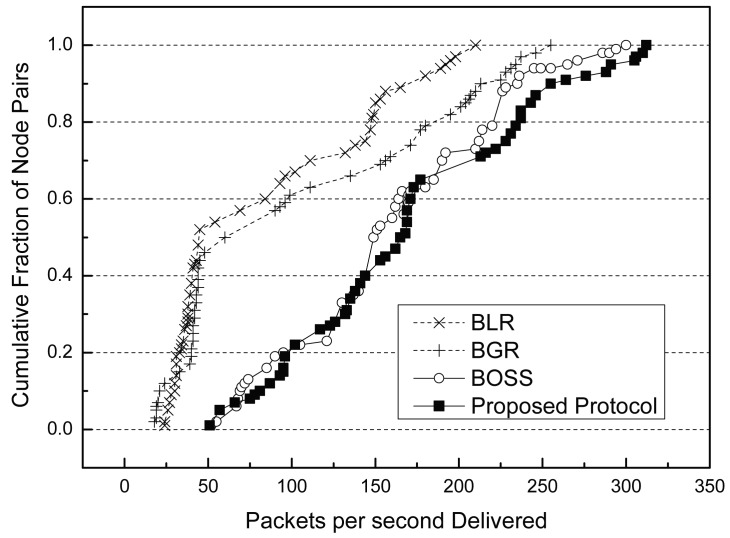
Data throughputs CDFs of 50 randomly-selected node pairs.

Figure 7 shows the distribution of throughputs of the three protocols. In this simulation, 500 sensor nodes are randomly deployed in the sensor fields. Each curve shows the throughput Cumulative Distribution Function (CDF) of the geographic routes for the same 50 randomly-selected node pairs. The *x* value of any point indicates throughputs in packets per second. The size of a packet is 100 bytes. The *y* value indicates what fraction of pairs has fewer throughputs.

The left two curves are the throughput CDFs achieved by using traditional beaconless routing protocols, BLR and BGR. The right two curves are the throughput CDFs achieved by BOSS and the proposed protocol, respectively. As shown in Figure 7, the proposed protocol provides almost three-times as much throughput as BLR and BGR for the median pair. This is because they have more routing failures than our protocol. In these protocols, only a few neighbor nodes could get a chance to be the next forwarding node. Unfortunately, this routing failure is related to the performance of the data throughput, because of unnecessary time loss. Every beaconless routing protocol has its own functional timer in order to be selected as an appropriate next hop forwarding node among neighbor nodes (for the contention process). This means that if routing fails in a hop, it has to start from the beginning, including the waiting time for the competition. For example, as shown in the previous Figure 1, the sender *S* can realize the routing failure only after the contention process is done. Namely, if *T_max_* is set to 300 ms, *S* could rebroadcast after 300 ms. If the timer is long, it affects the data throughput more. For low-throughput pairs, BLR and BGR have similar graphs. Because their processes are almost alike, however, since BGR exploits an improved recovery strategy, unlike BLR, it shows better performance than BLR. BOSS shows a similar graph as the proposed protocol. This is because both BOSS and the proposed protocol could use the full greedy area on every intermediate node during the routing process. However, the proposed protocol shows better performance than BOSS in most cases, due to the static contention time of BOSS.

### 4.3. Impact of the Number of Sensor Nodes

We evaluate the hop counts, the total number of packets, the total number of perimeter packets, the packet delivery ratio and the end-to-end delay for different numbers of sensor nodes, from 200 to 900.

**Figure 8:**
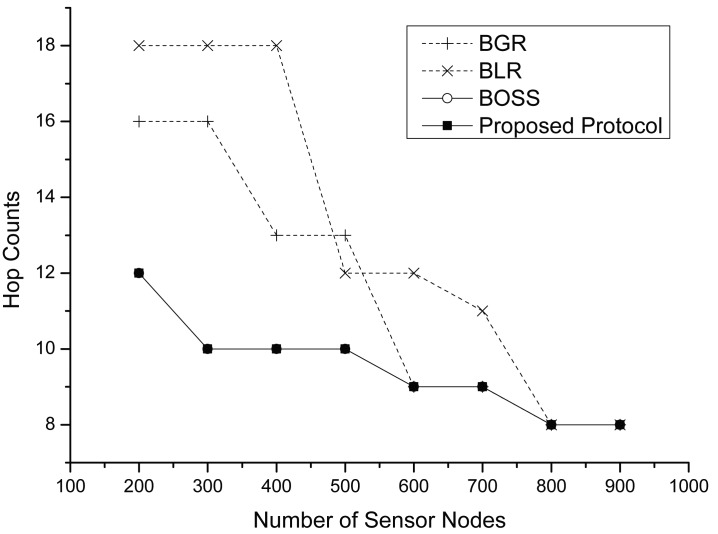
The hop counts vs. the number of sensor nodes.

Figure 8 illustrates a variation of the hop counts for each protocol. In both protocols, as the number of nodes increases, the average hop count decreases. When the node density increases, each node has more neighbor nodes than before. It results that each protocol could find a shorter route under high density conditions. We also observe that the proposed protocol and BOSS show a smaller number of hops than others when the number of sensor nodes is low. It is the case that only our protocol finds a geographic route in a hop, while the other protocols cannot find a route and misunderstand that there is no appropriate forwarding node. In this case, they should change their routing mode into perimeter mode. Therefore, they have a longer route than ours. However, if the node density increases, all protocols could have a higher probability of finding the next hop forwarder among mutually-communicable neighbor nodes. All protocols select the same neighbor as the next hop forwarding node; therefore, they have the same hop counts when there is high node density.

**Figure 9:**
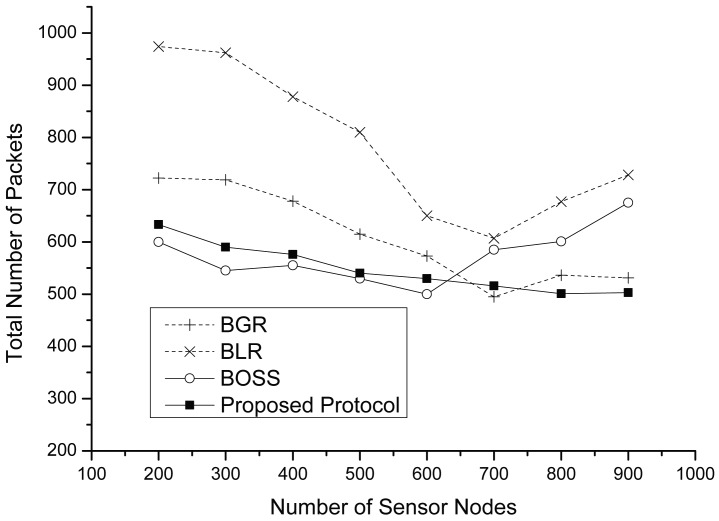
Total number of packets vs. the number of sensor nodes.

**Figure 10:**
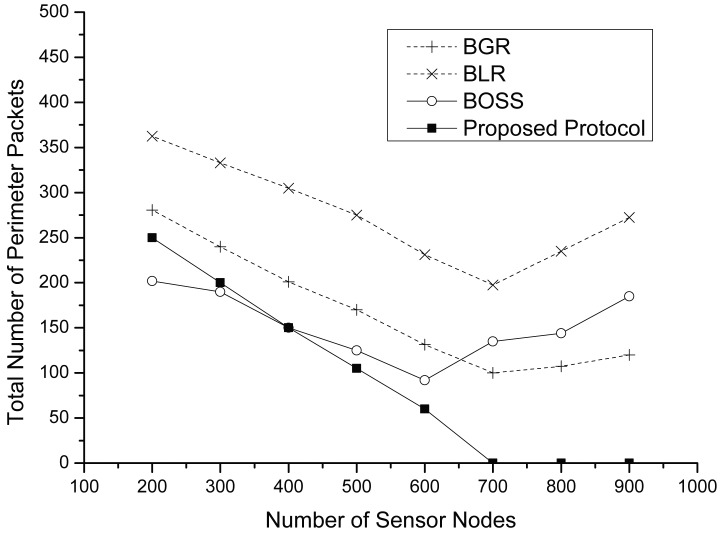
Total number of perimeter packets vs. the number of sensor nodes.

Figures 9 and 10 show the total number of packets and the total number of perimeter packets, including data and control packets, according to the variation of the node density. As shown in Figure 9, although the total number of packets of each protocol decreases before a 700-node density, in most cases, BLR and BGR have more packets than the proposed protocol. This is because they have more routing failures than the proposed protocol. However, the graphs of both BLR and BGR rapidly decrease at increasing densities. We observe that the path of each protocol is almost similarly constructed at a density of 700. Furthermore, in this graph, we can see another fact: that the total number of packet from both BLR and BGR is slightly increasing after a density of 700. The reason is that if the node density is extremely high, this might lead to the collisions produced by neighbors forwarding the message, because the timers expire almost concurrently. However, since the proposed protocol exploits a novel collision avoiding scheme, called DACAS, it has only a few collisions. When there is a low density, BOSS shows the best performance compared to the others. However, as the number of nodes increases, BOSS generates more packets. This is because CTS collisions among a number of neighbor nodes may generate a forwarding failure, thus leading to retransmission on the hop.

As shown in Figure 10, we figure out the total number of perimeter packets in detail. Generally, the proposed protocol shows a lower number of perimeter packets than others. Every protocol changes its routing mode into perimeter mode when all forwarding candidates cannot receive a data packet. Since both BLR and BGR have a lower number of forwarding candidate nodes than the proposed protocol, they have more of a chance to get changed into the perimeter mode. We observe that the proposed protocol has no perimeter packets after a 700-node density. Because, although there exist collisions in the high prioritized region due to extremely high node density, the rest of the nodes in the low prioritized region can relay data safely. On the other hand, the total number of perimeter packets of both BLR and BGR increases after a 700-node density. As mentioned above, the main reason is collisions due to high node density. In the case of BOSS, perimeter packets are generated by collisions among a high number of control packets. When there is high density, BOSS tends to forward data packets by recovery routing, although there exist appropriate nodes in the greedy area, since an intermediate sender cannot receive any CTSs from neighbors due to their own collisions.

**Figure 11:**
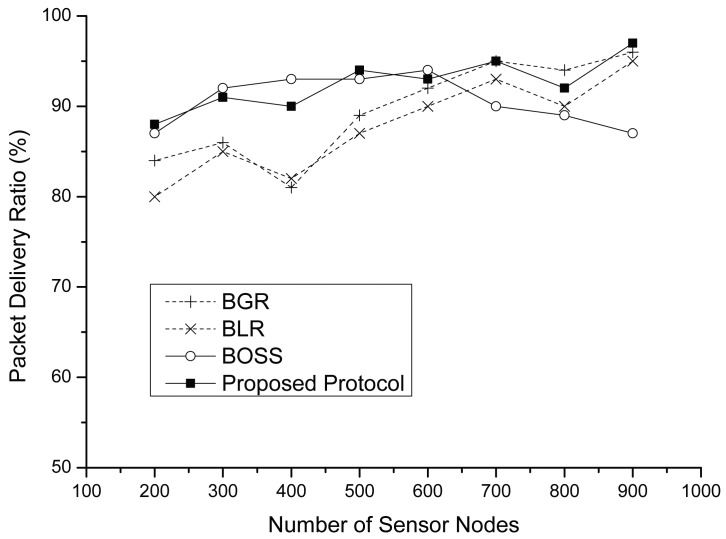
The packet delivery ratio vs. the number of sensor nodes.

Figure 11 shows the packet delivery ratio (PDR) among the four different protocols. As the node density increases, the PDR of each protocol also increases. This is because each protocol has a shorter path as the node density increases, as shown in Figure 8. Therefore, we can find that PDR is strongly related to the number of hops. In general, the proposed protocol shows higher PDR than BLR and BGR. The reason is that our protocol fully uses a forwarding candidate area, like the beacon-based protocols; however, other protocols use only a limited area that consists of mutually-communicable sensor nodes. These protocols can guarantee desired data throughputs for the destination only when the network has enough sensor nodes. On the other hand, BOSS shows a similar performance as the proposed protocol; however, this decreases when there is a high node density, due to the previously mentioned collision problem.

**Figure 12:**
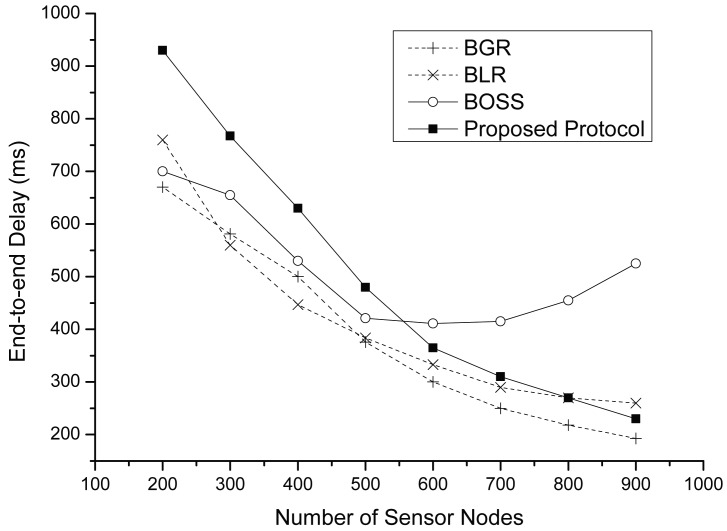
The end-to-end delay vs. the number of sensor nodes.

Figure 12 shows the end-to-end delay for each protocol. Except the case of BOSS, the graph shows that the density is strongly correlated with the end-to-end delay; because BOSS requires full-contention time whenever a packet is forwarding to a next-hop node. In the case of other protocols, the lower densities show longer end-to-end delay. In fact, routing in perimeter mode is the main source of long delays. Similarly, in this simulation, a large part of the end-to-end delay is from perimeter routing. However, although the proposed protocol has smaller perimeter packets than other protocols, we observe that the proposed protocol has more delay than the others with low densities. This is the case when there are no appropriate forwarding candidates in the greedy area, including both the mutually-communicable area and the rest of the area. In this case, both BLR and BGR turn into perimeter mode after *T_max_* (ms); however, the proposed protocol requires 2 × *T_max_* (ms) because of its two-phase contention process. However, as the node density increases, each protocol can find a similar route, and then, the end-to-end delay of each protocol also becomes short.

## 5. Conclusions

In this paper, we proposed a region-based collision avoidance beaconless geographic routing protocol that can be used in irregular wireless sensor networks. It aims at increasing forwarding opportunities by giving different contention priorities to the mutually-communicable nodes and the rest of the nodes in the greedy area. Our various experimental results showed that the proposed protocol has better performance than the previous protocols, as we intended.
